# Feasibility, Usability and Acceptability of a mHealth Intervention to Reduce Cardiovascular Risk in Rural Hispanic Adults: Descriptive Study

**DOI:** 10.2196/40379

**Published:** 2022-12-23

**Authors:** Sheri Rowland, Athena K Ramos, Natalia Trinidad, Sophia Quintero, Rebecca Johnson Beller, Leeza Struwe, Bunny Pozehl

**Affiliations:** 1 College of Nursing University of Nebraska Medical Center Lincoln, NE United States; 2 College of Public Health University of Nebraska Medical Center Omaha, NE United States; 3 College of Nursing University of Nebraska Medical Center Omaha, NE United States

**Keywords:** mHealth, health behavior, self-management, Hispanic/Latino, rural, apps, feasibility, acceptability, participation, engagement, wearable device, tracking, smartphone

## Abstract

**Background:**

Mobile health (mHealth) technology using apps or devices to self-manage health behaviors is an effective strategy to improve lifestyle-related health problems such as hypertension, obesity, and diabetes. However, few studies have tested an mHealth intervention with Hispanic/Latino adults, and no studies were found testing mHealth with rural Hispanic/Latino adults, the fastest-growing population in rural areas.

**Objective:**

The purpose of this study was to evaluate the feasibility, usability, and acceptability of an mHealth cardiovascular risk self-management intervention with rural Hispanic/Latino adults.

**Methods:**

A descriptive study using quantitative and qualitative methods was used to evaluate the feasibility, usability, and acceptability of delivering a 12-week mHealth self-management intervention to reduce cardiovascular risk with rural Hispanic/Latino adults who were randomized to 1 of 2 groups. Both groups were asked to use MyFitnessPal to self-monitor daily steps, weight, and calories. The intervention group received support to download, initiate, and troubleshoot technology challenges with MyFitnessPal (Under Armour) and a smart scale, while the enhanced usual care group received only a general recommendation to use MyFitnessPal to support healthy behaviors. The usability of MyFitnessPal and the smart scale was measured using an adapted Health Information Technology Usability EvaluationScale (Health-ITUES). Adherence data in the intervention group (daily steps, weight, and calories) were downloaded from MyFitnessPal. Acceptability was evaluated using semistructured interviews in a subsample (n=5) of intervention group participants.

**Results:**

A sample of 70 eligible participants (enhanced usual care group n=34; intervention group n=36) were enrolled between May and December 2019. The overall attrition was 28% at 12 weeks and 54% at 24 weeks. mHealth usability in the intervention group increased at each time point (6, 12, and 24 weeks). Adherence to self-monitoring using mHealth in the intervention group after week 1 was 55% for steps, 39% for calories, and 35% for weights; at the end of the 12-week intervention, the adherence to self-monitoring was 31% for steps, 11% for weight, and 8% for calories. Spikes in adherence coincided with scheduled in-person study visits. Structured interviews identified common technology challenges including scale and steps not syncing with the app and the need for additional technology support for those with limited mHealth experience.

**Conclusions:**

Recruitment of rural Hispanic/Latino adults into the mHealth study was feasible using provider and participant referrals. The use of MyFitnessPal, the smart scale, and SMS text messages to self-monitor daily steps, weights, and calories was acceptable and feasible if technology support was provided. Future research should evaluate and support participants’ baseline technology skill level, provide training if needed, and use a phone call or SMS text message follow-ups as a strategy to minimize attrition. A wearable device, separate from the smartphone app, is recommended for activity tracking.

## Introduction

### Background

Mobile health (mHealth) refers to the use of mobile and wireless technology to achieve a health objective. While there is no standard for what constitutes mHealth, common features used alone or in combination include smartphone apps, SMS text messaging, and wearable monitoring devices. mHealth interventions targeting cardiovascular health are effective in supporting self-management of glycemic control [1,2], blood pressure [3], weight [4], and physical activity behavior [5]. Despite the evidence of mHealth intervention effectiveness, few studies have included Hispanic/Latino adults [6,7], and no studies focused on rural-living Hispanic/Latino adults were found [1]. In urban-living Hispanic/Latino adults with type II diabetes, mHealth improved medication adherence, increased fruit and vegetable consumption, and increased physical activity with SMS text messages 3 times a day for 3 weeks [8]. Another study reported lowered hemoglobin A_1c_ (HbA_1c_) levels by sending SMS text messages (informational, motivational, and prompting) over 6 months [2].

Hispanic/Latino adults, a population underrepresented in mHealth intervention studies targeting cardiovascular risk, have notable cardiovascular health disparities. In the general population, type II diabetes affects 17% of Hispanic/Latino adults compared to 8% of non-Hispanic White adults [9]. Cardiovascular morbidity and mortality outcomes are worse for the Hispanic/Latino adult population with a greater risk of developing heart failure [10] and a 50% higher likelihood of death from complications related to diabetes than non-Hispanic White adults [11]. Among Hispanic/Latino adults living in rural areas, the prevalence of obesity is higher (Hispanic/Latino adults: 36%; non-Hispanic White adults: 32%), and health status is reported as low or poor (Hispanic/Latino adults: 28%; non-Hispanic White adult: 19%) [12]. Healthy diet and physical activity behaviors, which can be self-managed using mHealth, are less common among Hispanic/Latino adults, particularly among those with type II diabetes [13,14]. Clearly, the Hispanic/Latino adult population has increased cardiovascular risk that is not adequately managed.

An estimated 19% of the total Unites States population is Hispanic with the fastest growth of this population observed in rural areas [15]. In the United States, an estimated 79% of Hispanic/Latino adults and 71% of rural-living adults own a smartphone [16]. Most adults in rural areas access the internet daily (76%); however, high-speed internet access is a major concern for rural adults (24%) compared to urban adults (13%) [17]. While Hispanic/Latino adults are noted to use smartphones to text and access email [18], it remains unclear how likely they are to use smartphone apps to actually manage their health [19]. One study in rural-living Hispanic/Latino adults indicated that 81% had the willingness to use mHealth, but few (15%) were aware of how mHealth could support the self-management of chronic health conditions like hypertension and diabetes [20]. A national survey on health app use found that Hispanic/Latino adults were significantly more likely to use a health app compared to non-Hispanic adults, and the most common feature desired was the ability to communicate with a health care provider through the health app [21].

### Theoretical/Conceptual Framework

The intervention in this pilot study was based on the concept of chronic disease self-management. Specific self-management skills include decision-making (what is my priority health problem?), problem-solving (what can I do to manage this problem?), action-taking (how do I begin managing this problem?), accessing resources, and partnering with a health care provider [22]. Goal setting theory [23] and self-efficacy, a concept of social cognitive theory [24], support the mHealth intervention to build self-management skills and establish new patterns of healthy behavior (Textbox 1).

**mHealth intervention components.**

**Goal setting**

- Priority health conditions selected
  Participant and nurse practitioner (NP) determine together
- Investigator-set participant goals
  Weigh daily using scale synced to MyFitnessPal (MFP)
  Log all food and drink consumed in MFP
  Achieve prescribed daily calorie target based on age, gender, and activity level [25]
  Walk 10,000 steps per day using MFP step tracker

NP visit

- Baseline-15 min
- 6 weeks-15 min
- 12 weeks-15 min

**Self-efficacy**

- Physiologic feedback
  Biometrics, lab tests, and fitness test performed
  Results reviewed and personalized report given to the participant

NP visit

-
  Baseline-15 min
  6 weeks-15 min
  12 weeks-15 min
- Verbal persuasion

SMS text messages to prompt MFP use, provide health information on priority health conditions, and encouragement

- Twice weekly via Remind smartphone app
- Mastery experiences
  Use digital scale to see sync to MFP
  Find daily step count in MFP
  Practice logging food/drink items in MFP
- Tech support visit
  Baseline-30 min
  6 weeks-15 min
  12 weeks-15 min

### Purpose

The purpose of this study was to evaluate the feasibility, usability, and acceptability of a stand-alone mHealth cardiovascular risk self-management intervention using MyFitnessPal, a compatible smart scale, and an engaged bilingual health care provider in a rural Hispanic/Latino adult population.

Given the limited number of mHealth interventions to reduce cardiovascular risk in the Hispanic/Latino adult population, it is important to understand what aspects of mHealth interventions are most engaging and usable.

## Methods

### Study Design

A descriptive study design using quantitative and qualitative methods was used to evaluate the feasibility, usability, and acceptability of a cardiovascular risk self-management mHealth intervention conducted with rural Hispanic/Latino adults between May 2019 and June 2020. The 12-week intervention was tested using an unblinded randomized 2-group design. Both groups were asked to use MyFitnessPal to self-monitor daily steps, weight, and calories. The intervention group received support to download, initiate, and troubleshoot technology challenges with MyFitnessPal and a smart scale, while the enhanced usual care group received only a general recommendation to use MyFitnessPal to support healthy behaviors. This report details the feasibility and acceptability of the mHealth intervention and compares mHealth usability between the 2 groups. The cardiometabolic health outcomes in both groups are reported separately [26].

### Sample and Setting

A sample of 70 rural-living Hispanic/Latino adults was recruited from 2 rural communities in Nebraska. Inclusion criteria were Hispanic/Latino adults (aged 19-65 years); English or Spanish speaking; living within 50 miles of either community; self-report hypertension, obesity, type II diabetes, or dyslipidemia; having a smartphone; and using Bluetooth or Wi-Fi. Exclusion criteria were current participation in a health behavior program (eg, Weight Watchers), BMI≥45 unless a clinician granted permission, or high risk for a cardiac event during fitness testing. Pregnant women were excluded from the step fitness test, BMI, and waist measurement.

In this study, a minimum sample of 54 (27 per group) was determined by setting the desired margin of error for the mean metabolic equivalents (METs), the primary outcome of cardiorespiratory fitness, at no more than 0.80. Using a standard deviation for walking METs of 2.00, a minimum sample size of 27 per group yields a margin of error of 0.75. A margin of error assumed a 95% confidence interval. With an assumed attrition rate of 30%, the total sample goal was 70 (35 per group), which is appropriate for pilot work, allowing for descriptive statistics, estimation of effect sizes, and hypothesis generation [27].

### Intervention Development

To support intervention development and delivery, a community advisory board was established. Members of the board included two Hispanic/Latino community residents and representatives of the local federally qualified health clinic, local public health department, Hispanic community center, and educational service unit. The board had 2 in-person meetings and communicated through email to advise on community resources, recruitment strategies, and the cultural relevancy of the SMS text messages that were developed for the intervention group.

#### mHealth Intervention Group

The intervention group received the MyFitnessPal premium version (US $50), the Withings Body+ smart scale (US $75), consultation with a bilingual nurse practitioner and tech support person, and SMS text messages twice a week via the free Remind app in the participant preferred language (English or Spanish) (Textbox 1). MyFitnessPal premium was selected because of the availability in Spanish, the large food database (6 million items), and the capacity to track adherence through a “diary share” feature and a downloadable CSV data file. Bilingual tech support was provided to initialize all technology including assisting with downloading and setting up the MyFitnessPal app on participants’ phones. During the 30-minute tech support visit, each participant received verbal and written instructions on how to use both the app and the smart scale. The teach-back method was used to confirm understanding.

#### Enhanced Usual Care Group

This group received a general recommendation to use the free version of MyFitnessPal to support a healthy lifestyle, but they did not receive technical support to download or initiate the app. At the 6-week visit, the participants had a weight and blood pressure check and were again encouraged to use MyFitnessPal but were not provided technical support. At the last 24-week visit, this group received the smart scale, a consultation with the bilingual nurse practitioner, and a visit with a tech support person.

### Procedures

To standardize the intervention the following were used: a training manual with topic checklist, set time allowances for study activities, a script for participant visits, and role-playing participant visits prior to beginning recruitment. Participants were recruited using bilingual flyers posted in the community, zip code–targeted Facebook ads, and within the local clinic. A study coordinator took calls from those interested in the study, discussed eligibility, and scheduled study visits at either the mobile clinic or the local community center. Following informed consent and baseline measures, the participants were randomized to either the enhanced usual care group or the intervention group. Both groups had visits at baseline, 12 weeks, and 24 weeks to complete paper questionnaires, collect lab tests (HbA_1c_ and lipids), measure biometrics (blood pressure, BMI, and waist circumference), and complete a 2-minute step test to evaluate cardiorespiratory fitness. At a 6-week booster visit, weight and blood pressure were measured. Study visits were conducted in a reserved area of a community center, a conference room in a large family practice clinic, or in a small community clinic during off-clinic hours.

#### Ethics Approval

The study was approved by the institutional review board at the University of Nebraska Medical Center (reference number: 26). Informed consent was completed in person and in the language preferred by the participant (English or Spanish). Personal identifying information collected was limited to name, postal address, date of birth, and telephone number, and these data were stored in a locked file box and on a secure, password-protected server. Participant data were collected using paper instruments identified with a unique identification number. Participants were given a US $25 gift card for each completed visit.

### Measures

#### Usability

An adapted Health Information Technology Usability Evaluation Scale (Health-ITUES) was used to evaluate the usability of the MyFitnessPal app and compatible smart scale. This 20-item tool uses a 5-point Likert scale (strongly disagree to strongly agree) across 4 subscales: impact on daily life, perceived usefulness, perceived ease of use, and user control [28]. Examples of items for each of the subscales include, “I think MyFitnessPal and smart scale would improve the quality of life for a person living with chronic cardiovascular conditions,” “Using MyFitnessPal and smart scale makes easier to self-manage my cardiovascular-related conditions,” “I find MyFitnessPal premium and smart scale easy to use,” and “Whenever I make a mistake while using MyFitnessPal and smart scale, I recover easily and quickly.” The scale has an overall mean possible score range of 20-100 with higher scores indicating higher usability. In our study, the overall scale had excellent reliability, Cronbach α=.94. Subscales also had good reliability with Cronbach α ranging from .82 to .97. Participants completed this measure in paper form in their preferred language (Spanish or English). The adapted Health-ITUES was completed by all intervention group participants at 6, 12, and 24 weeks. In the enhanced usual care group, only those who reported downloading/using MyFitnessPal were asked to complete the adapted Health-ITUES at 6, 12, and 24 weeks.

#### Adherence

Adherence was only measured in the intervention group because only the premium version of MyFitnessPal allows access to usage data. An Excel file reporting on steps (daily total), weight (by date), and calories (data total) was accessed for each intervention group participant.

#### Steps

Participants were asked to carry their phones with them throughout the day to track steps using the MyFitnessPal step tracker and work toward 10,000 steps per day. The step tracker is an automatic feature that was turned on at the first tech support visit.

#### Weight

Participants were asked to weigh themselves naked each morning using the scale that was synced to MyFitnessPal at the first tech support visit. Participants were shown how to manually enter a weight into MyFitnessPal in the event an automatic sync did not occur.

#### Calories

Participants were asked to log all food and nonwater beverages consumed each day using the MyFitnessPal diet tracker. They were also asked to work toward achieving a daily calorie limit based on their age, gender, and activity level [25].

### Acceptability

A stratified sampling approach was used to select intervention group participants for a 15-minute semistructured interview on the acceptability of mHealth upon completion of the intervention. Interviewees were selected based on gender (male/female), age (under/over 40), and level of engagement during the study (high/low). Participants were asked specifically about their experience with (1) MyFitnessPal to track steps and calories, (2) the smart scale to track daily weight, and (3) twice weekly SMS text messages. The interviews were conducted face-to-face in a private area of the data collection space (conference room and clinic room) in the language preferred by the participant.

### Analysis

Quantitative data were examined for missingness, errors, normality, and equality of variances. Continuous variables were analyzed using means and standard deviations. Categorical variables were analyzed using frequencies and percentages. Only participants who responded to a measure at a specific time point were included in analyses; therefore, the number reported at each time point varied. Qualitative data were collected through audiorecorded interviews that were transcribed verbatim in Spanish and then translated to English for thematic analysis [29].

## Results

### Feasibility

During enrollment (May-December 2019), 77 people expressed interest in participating, but 7 were not enrolled because they lacked a qualifying health condition. Of the 70 participants who enrolled, 45 were referred by someone already in the study, and 25 were referred by their health care provider. Table 1 details the sample demographic characteristics by group. Group differences in the demographic data were assessed with independent *t* tests, chi-square tests, and Fisher exact tests. No demographic variables were significantly different except for the preferred language in the study, where 77% (26/34) of the enhanced usual care group and 94% (34/36) of the intervention group preferred to complete the study in Spanish (questionnaires, informational SMS text messages, and MyFitnessPal; Fisher exact test *P*=.04).

The overall attrition was 28% (n=19) at 12 weeks and 54% (n=38) at 24 weeks. Attrition by group was similar at 12 weeks (enhanced usual care: n=9, 26%; intervention: n=11, 31%) and 24 weeks (enhanced usual care: n=18, 53%; intervention: n=21, 57%). Although there was missing data, this was primarily due to physical limitations with biometric testing, a mechanical limitation of blood analyzer (result too high to read), unanswered questions, and suspension of in-person data collection due to COVID-19 during the last 2 months of the study. Instead, phone calls were made to 15 participants to collect responses to the questionnaires and a self-reported weight.

**Table 1:** Baseline participant characteristics by group.

		Enhanced usual care group (n=34)			Intervention group (n=36)	
		Responders, n	Value	Responders, n		Value
Age (years), mean (SD)		34	40.8 (9.9)	36		41.4 (9.8)
Female, n (%)		34	23 (68)	36		31 (86)
Married/cohabitating, n (%)		33	27 (82)	35		29 (83)
**Education, n (%)**						
	Less than 12th grade	34	21 (58)	36		16 (47)
	More than 12th grade	34	15 (44)	36		18 (50)
**Country of origin, n (%)**						
	United States	34	9 (26)	35		7 (19)
	Mexico	34	21 (62)	35		19 (53)
	Guatemala	34	1 (3)	35		4 (11)
	El Salvador	34	2 (6)	35		3 (8)
	Honduras	34	1 (3)	35		2 (6)
**Language, n (%)**						
	English proficient	33	11 (33)	35		9 (26)
	Preferred Spanish during the study	34	26 (77)	36		34 (94)
**Household income per year (US $), n (%)**						
	<10,000	33	8 (24)	35		6 (17)
	10,000-50,999	33	23 (68)	35		22 (61)
	≥60,000	33	2 (6)	35		3 (8)
	Unemployed	33	16 (47)	35		17 (47)
**Number of people living in a household, n (%)**						
	1-3	34	5 (15)	33		7 (19)
	4	34	15 (44)	33		13 (36)
	≥5	34	14 (41)	33		13 (36)
No health insurance, n (%)		33	18 (53)	36		18 (50)

### Usability

Usability was higher in the intervention group at all 3 time points and increased at each time point (6, 12, and 24 weeks). Fewer participants in the enhanced usual care group completed the usability measure compared to the intervention group at all 3 time points. This was due to only collecting the Health-ITUES from those who reported downloading the free version of MyFitnessPal (Table 2). Given the small numbers in the enhanced usual care group, descriptive statistics are reported rather than a statistical test of difference between groups.

**Table 2:** Perceived usability of mHealth by groups.

			Enhanced usual care^a^ (n=34)									Intervention (n=36)						
			Responders, n		Min		Max		Score, mean (SD)		Responders, n			Min		Max		Score, mean (SD)
**At 6 weeks**																		
	Impact on daily life	3		6		15		9.33 (4.93)		24			5		15		12.71 (3.06)	
	Usefulness	3		18		45		27.33 (15.31)		24			18		45		35.92 (9.36)	
	Ease of use	3		14		25		21.33 (6.35)		23			11		25		20.43 (5.09)	
	User control	3		7		15		11.33 (4.04)		24			3		15		11.50 (3.74)	
	Composite score	3		45		100		69.33 (28.04)		21			56		100		82.29 (15.47)	
**At 12 weeks**																		
	Impact on daily life	8		11		15		13.50 (1.69)		23			9		15		13.78 (2.087)	
	Usefulness	8		27		45		36.75 (6.39)		22			24		45		40.45 (7.29)	
	Ease of use	8		15		25		22.50 (3.54)		23			7		25		21.26 (5.21)	
	User control	8		8		15		12.38 (2.77)		23			7		15		12.69 (2.82)	
	Composite score	8		66		100		85.13 (12.30)		22			48		100		88.23 (15.64)	
**At 24 weeks**																		
	Impact on daily life	6		11		15		12.83 (1.72)		22			11		15		13.91 (1.44)	
	Usefulness	6		31		45		36.67 (6.59)		20			31		45		42.10 (4.22)	
	Ease of use	6		20		25		22.67 (2.58)		22			12		25		21.59 (4.35)	
	User control	6		9		15		11.83 (2.04)		21			6		15		11.91 (3.22)	
	Composite score	6		76		100		84.00 (9.44)		19			71		100		90.95 (10.48)	

^a^Only those who reported downloading MyFitnessPal were asked to complete usability measure.

### Adherence

Intervention group adherence data were obtained from the MyFitnessPal premium app, which reported a daily step total, weight by date, and a daily calorie total. A dichotomous variable was created to indicate the presence or absence of captured data for each of the self-monitoring activities: daily steps, weight, and calories/meal (breakfast, lunch, and dinner). Weekly adherence for steps, weight, and calories was determined by the number of days per week of data capture. In this study, participants were adherent to mHealth self-monitoring if there was data captured ≥4 days per week for each week during the intervention (weeks 1-12) and postintervention (weeks 13-24).

#### Steps

**Figure 1:**
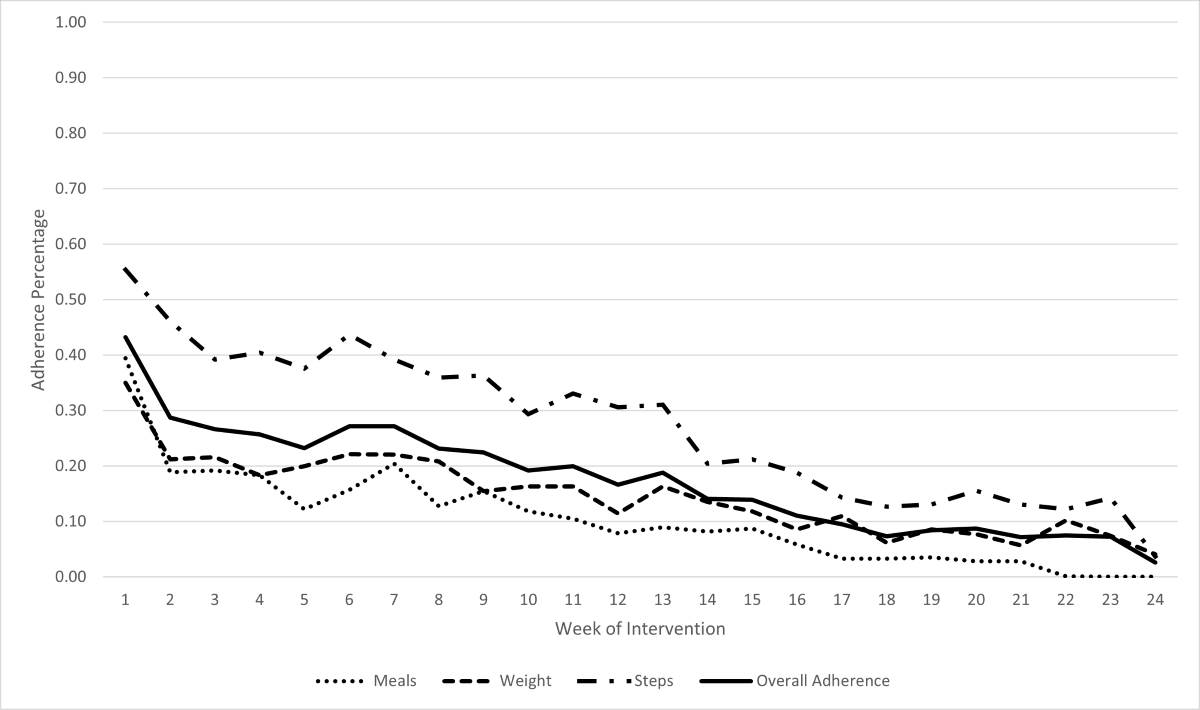
Percentage of intervention group participant adherence.

In the first week of data collection, 55% (n=20) of the intervention group participants were adherent to the daily step capture using MyFitnessPal premium. Step adherence fell gradually to 31% (n=11) at the end of the intervention at week 12. Step adherence continued to gradually decline to 0% at 24 weeks. An observable spike in step adherence was noted at weeks 6 and 11 (Figure 1). The increases in step adherence coincide with in-person study visits at weeks 6 and 12.

#### Weights

In the first week of data collection, 35% (n=12) of the intervention group participants were adherent to the daily weight capture using MyFitnessPal premium and compatible smart scale. Weight adherence trended down to 11% (n=4) at the end of the 12-week intervention and to 0% at week 24. Similar to step adherence, there was a spike in weight adherence at weeks 5 and 11, which corresponds with in-person study visits at weeks 6 and 12, respectively.

#### Calories

In the first week of data collection, 39% (n=14) of the intervention group participants were adherent to logging food and nonwater beverages consumed each day using the MyFitnessPal diet tracker. Food logging trended down to 8% (n=3) for the intervention group participants at week 12 and to 0% at 24 weeks.

### Acceptability

Five participants completed an interview about the intervention. Three interviewees were low engagers (2 female participants and 1 male participant) and 2 were high engagers (1 female participant and 1 male participant). Three themes were identified: (1) mHealth is useful, (2) mHealth has challenges, and (3) changes may be needed for success with a future study (Textbox 2). Participants described mHealth as useful by creating “self-awareness,” “personal accountability,” “empowerment,” and “motivation” to work on their health behaviors. The Spanish setting in MyFitnessPal and Spanish SMS text messages were appropriate and understandable. One participant remarked, “it [mHealth] works not only for you but for the other members of your family.” Participants also described exploring and using other mHealth features including macronutrient information, BMI calculation, and the visual weight trend on the scale.

**Intervention group participant quotes.**

**Mobile health (mHealth) is useful**

- “I was in control of my food, I was able to register it, and I was able to give myself feedback with regard to what I was doing. I was able to see it there, in real time or what I was eating, if I was losing [weight], if I was doing enough exercise,” (ID 6)
- “It helps me control my food intake because I can see that I am close to reaching my daily goal.” (ID 2)
- “They [text messages] make me feel important. And also, it motivates me to keep putting in the effort.” (ID 49)
- “You notice whether or not you exercised that day...if I was not able today, I can do it tomorrow.” (ID 40)

**mHealth has challenges**

- “It [MyFitnessPal] did not have the data with typical Latino foods…. For example, there was fried Asian rice, but it is not the same as fried Latino rice.” (ID 6)
- “Registering the foods…I think it is harder to do because we are Latinos and we cook in large quantities and we serve many portions.” (ID 13)
- “It is a bit complicated to be able to register my foods.” (ID 49)
- “I always carry my phone with me, but I don’t always have the location on. Therefore, I knew that sometimes I personally had walked, let us say, 10,000 steps, but only 7,000 or 6,000 were registered —it did not help me to stay motivated because I would say to myself “I did so much, and it only registered 6,000 steps.” (ID 6)
- “I had to restart the scale five times along with my phone.” (ID 6)

**Recommendations for a future study**

- “You need to give yourself time to register or to learn because I really have not learned to do it well.” (ID 40)
- “Explain to us a bit more about the portions that we need to eat during the day.” (ID 40)
- “If I had had a different device, I think I would have liked it better. But to just use the phone to keep track of steps, no.” (ID 6)

Participants experienced disruptions in the automatic sync function of the app, resulting in steps and weights that were not captured. These technical issues would either be ignored, managed, or reported to the technology support person during in-person meetings. The accuracy was questioned by participants. They reported the counter was not picking up steps or they may not have had their phone with them when being active. The accuracy of calorie tracking was also questioned because portion size estimation was difficult, and the food database had limited ethnic foods. Three participants noted time as a barrier to logging food into the app.

The low mHealth engagers suggested more training or orientation for those who were not as skilled with smartphone use. Additional training was also suggested for entering recipes into MyFitnessPal so that homemade food could be accurately tracked. Most of the interviewees “liked” the SMS text messages and felt they were “good” and helpful to “remind me to do something for my health.” One participant did not find the SMS text messages “motivating” and suggested a feature to “opt-out” of receiving messages. A wearable activity monitor instead of the MyFitnessPal app was suggested to increase the accuracy of activity tracking.

## Discussion

To our knowledge, this is the first study to evaluate the feasibility, usability, and acceptability of a randomized mHealth cardiovascular risk self-management intervention with rural Hispanic/Latino adults. The feasibility of recruitment or enrollment was evaluated as adequate, and participant retention was evaluated as mixed. The low number of participants in the enhanced usual care group who downloaded and used the MyFitnessPal app as advised suggests that the mere recommendation by a health care provider to use a widely available commercial app to self-manage health behaviors is not enough to trigger and sustain mHealth use. Among those who received mHealth support in the intervention group, the usability of the MyFitnessPal app and smart scale to self-monitor daily steps, weight, and calorie intake was high and increased over time. Adherence to using the app and scale followed a typical gradual decline trajectory with upticks corresponding with in-person visits. Indications that the mHealth intervention was not acceptable were not detected in the subsample of intervention group participants who completed semistructured interviews.

Engaged health care providers and referrals from enrolled participants allowed the target sample of 70 participants to be enrolled over an 8-month period. For a larger, fully powered trial, it will be important to ensure buy-in and support of key local health care providers and consider incentivizing enrolled participants to refer others from their social network to participate. While the COVID-19 pandemic likely contributed to the high attrition rate at 24 weeks (n=38, 54%), the attrition rate at 12 weeks (n=19, 28%) was higher than the average rate of 18% observed in health behavior trials [30] but less than the 40% observed in app-based interventions targeting chronic disease management [31]. In a 24-week mHealth intervention targeting glycemic control in 126 urban-living Hispanic/Latino adults, 16% (n=11) of the intervention group and 5% (n=3) of the usual care group did not complete any follow-ups [2]. Participants presented for in-person data collection at baseline, 3 months, and 6 months; however, a study coordinator called the patient if a blood glucose reading exceeded a set range or if a blood sugar reading was not received for 1 week [2]. Phone calls, to build *personalismo*, may be a particularly culturally relevant strategy for reaching and maintaining contact with Hispanic study participants [32]. Future studies may consider the measurement and evaluation of *personalismo* in relation to mHealth engagement by Hispanic participants [32].

Increasing usability, as measured by the adapted Health-ITUES, over 3 time points within the intervention group is an indication that with time and experience participants become more comfortable, and therefore, mHealth may grow more useful for managing one’s own health. Difficulties with app use reported by low engagers during the structured interviews indicate a need to evaluate participants on their skill and confidence with mHealth technology prior to using this type of mHealth in the future. Even when participants owned a smartphone and used the SMS text messaging feature, it did not ensure competency with a commercially available health app like MyFitnessPal. Others have found that Hispanics may not have the knowledge to fully benefit from prevalent functionalities (ie, calorie tracker, macronutrient information, and sharing food logs with peers for accountability) offered by health apps [33]. An eHealth Literacy Assessment Toolkit is available, which includes reliable and valid methods of assessing not only health literacy but also technology familiarity, confidence, and incentives for engagement [34]. For future studies, assessing technology skills and confidence at the beginning of the intervention would allow for adjustment and tailoring of the mHealth training and orientation to better meet participant needs. In the enhanced usual care group, very few participants downloaded the free version of MyFitnessPal as advised. This limited the feasibility of comparing usability outcomes between groups, and future studies should consider alternative designs.

mHealth adherence in this study followed a trajectory similar to that observed in other intervention studies using a health app to self-monitor diet, weight, and activity in both rural and urban adults [35,36]. Specifically, adherence waned over the course of the study with sharper declines following the end of the intervention. In this study, adherence increases were observed around 6, 12, and 24 weeks when in-person visits occurred. It may be that the in-person visits reminded participants that they were being observed, and therefore, they altered their behavior. Group differences in the theoretical measures of self-management (self-efficacy, activation, and self-regulation) are reported in [26]. Although not significant, a medium intervention effect on self-regulation was observed at 12 and 24 weeks. In this study, the intervention group received in-person visits with a bilingual nurse practitioner and technology support person, which may have facilitated the development of self-monitoring skills (weight, calorie intake, and physical activity).

The qualitative structured interviews informed on acceptability of the mHealth intervention. While participants may have been reluctant to be overly critical of the study with the research team, indications of complete unacceptability were not detected. Low engagers acknowledged their own barriers to using the self-monitoring tools (MyFitnessPal app and smart scale) as well as provided suggestions on how to reduce barriers to mHealth usage. Acceptability may be enhanced by providing those with limited experience or low confidence with technology additional training, use of a wearable activity monitor instead of using an app to capture steps, training/troubleshooting on entering recipes into a food database, and culturally tailored education on portion size. Other methods of evaluating acceptability should be considered. For example, ecological momentary assessments (EMAs) provide a real-time data collection strategy delivered in the user’s natural environment. Among low-income Spanish-speaking adults, EMAs and language content analysis of brief SMS text message responses allowed for a deeper understanding of participant responses to depression treatment [37].

A limitation of this study was that adherence data were not collected in the enhanced usual care group, as the free version of MyFitnessPal does not have data extraction features. A future study would need to have adherence data collected from all participants to examine theoretical drivers of adherence. Adherence increases may also be explained by technology fixes that occurred during in-person visits such as re-establishing the capture of steps and scale and app syncing, although the increases are noted to begin the week prior to in-person visits. We acknowledge that the broad use of “Hispanic/Latino” does not recognize the full diversity of the population in the United States. Future studies should include additional demographic variables and a sample large enough to be able to denote specific subgroup differences.

Despite the increased use of mHealth in the United States, few culturally and linguistically tailored mHealth interventions have been tested with the Hispanic/Latino adult population. We found that a mHealth intervention using MyFitnessPal, a smart scale, and SMS text messages with rural Hispanic/Latino adults to self-monitor daily steps, weights, and calories was acceptable and feasible if technology support was provided. Like most mHealth interventions with predominately White populations, mHealth engagement with our Hispanic/Latino adult sample waned over time, and culturally tailored strategies to combat attrition are recommended. Professionals who are considering using mHealth technologies in practice should tailor them according to individual skill level, experience, and confidence. App developers should consider cultural and linguistic tailoring to meet the needs of diverse populations. In conclusion, mHealth can be leveraged to promote public health and help patients self-manage cardiovascular risk factors, particularly in the absence or limited supply of health care providers in rural communities.

## Abbreviations

EMA: ecological momentary assessment
HbA_1c_: hemoglobin A_1c_
Health-ITUES: Health Information Technology Usability Evaluation Scale
MFP: MyFitnessPal
mHealth: mobile health

## Data Availability

Access to raw data in .sav format is available upon request.
